# Supplementary figures and images for: Nucleosome breathing and remodeling constrain CRISPR-Cas9 function
Source: eLife. 2016 Apr 28;5:e13450. doi: 10.7554/eLife.13450 (PMC4880442; doi:10.7554/eLife.13450)

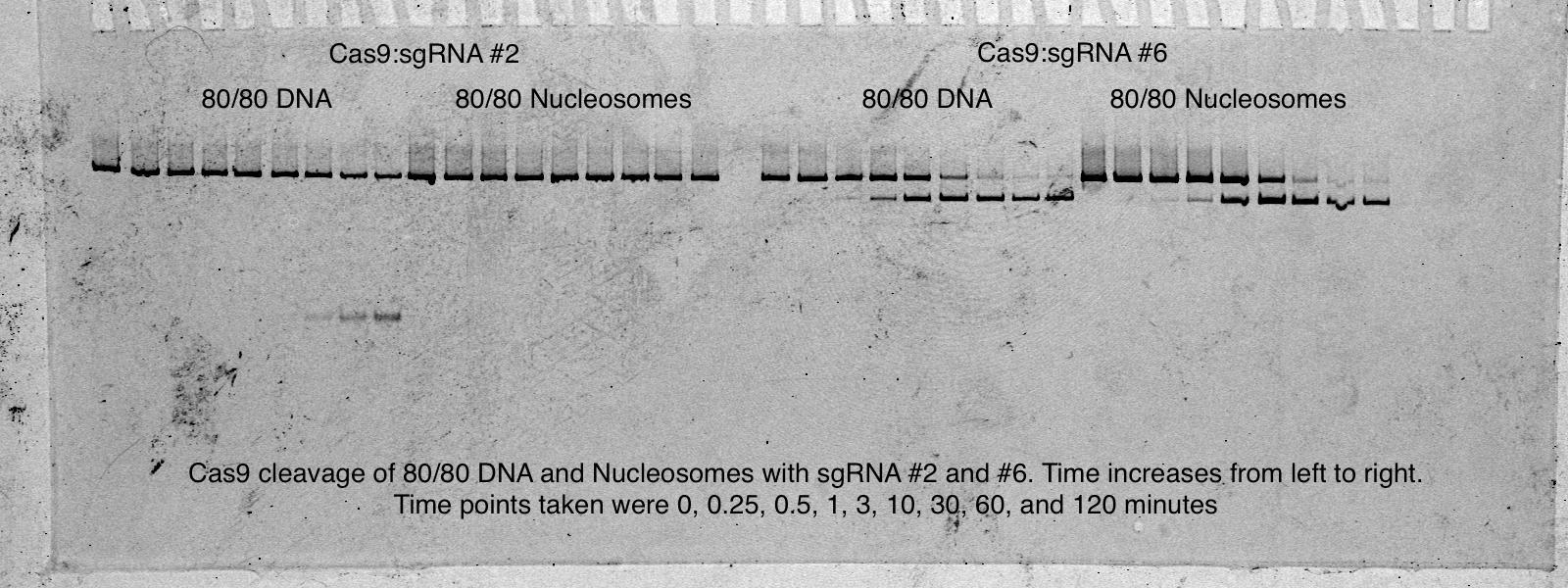

Supplement: Figure 1—source data 1. — DOI: http://dx.doi.org/10.7554/eLife.13450.004 [file elife-13450-fig1-data1.zip › Figure_1Source_Data_1.jpg]

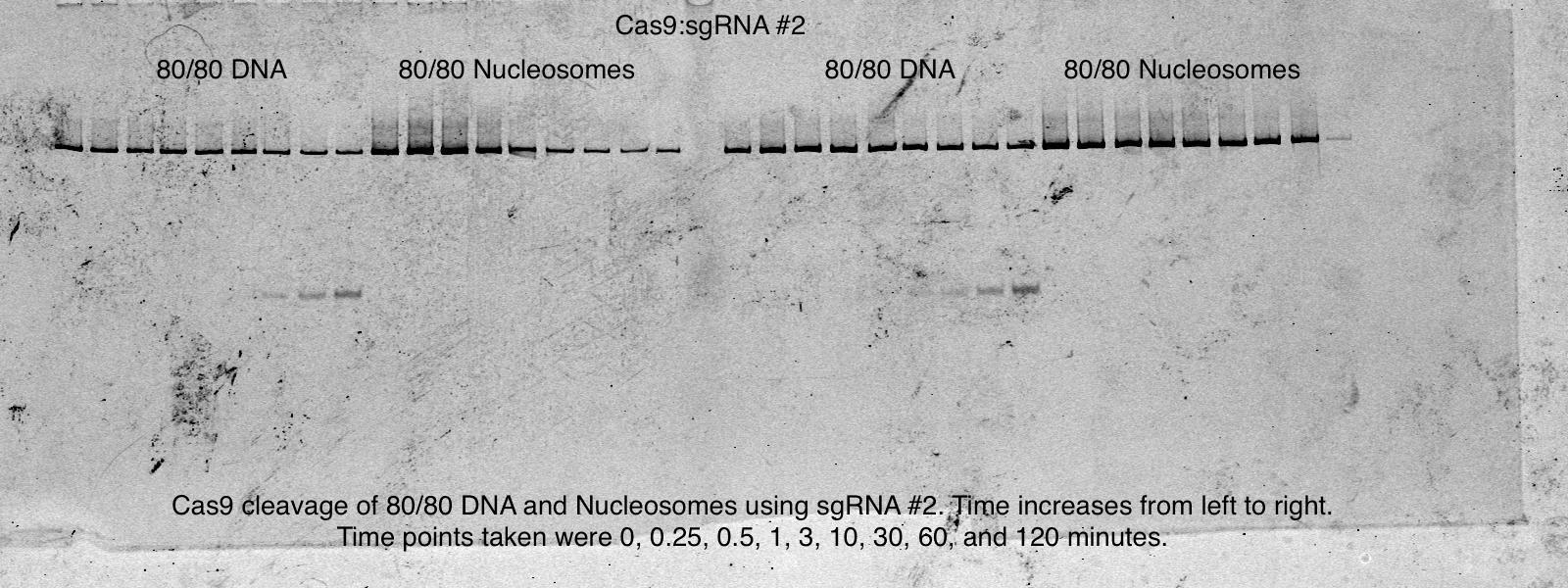

Supplement: Figure 1—source data 2. — DOI: http://dx.doi.org/10.7554/eLife.13450.005 [file elife-13450-fig1-data2.zip › Figure_1Source_Data_2.jpg]

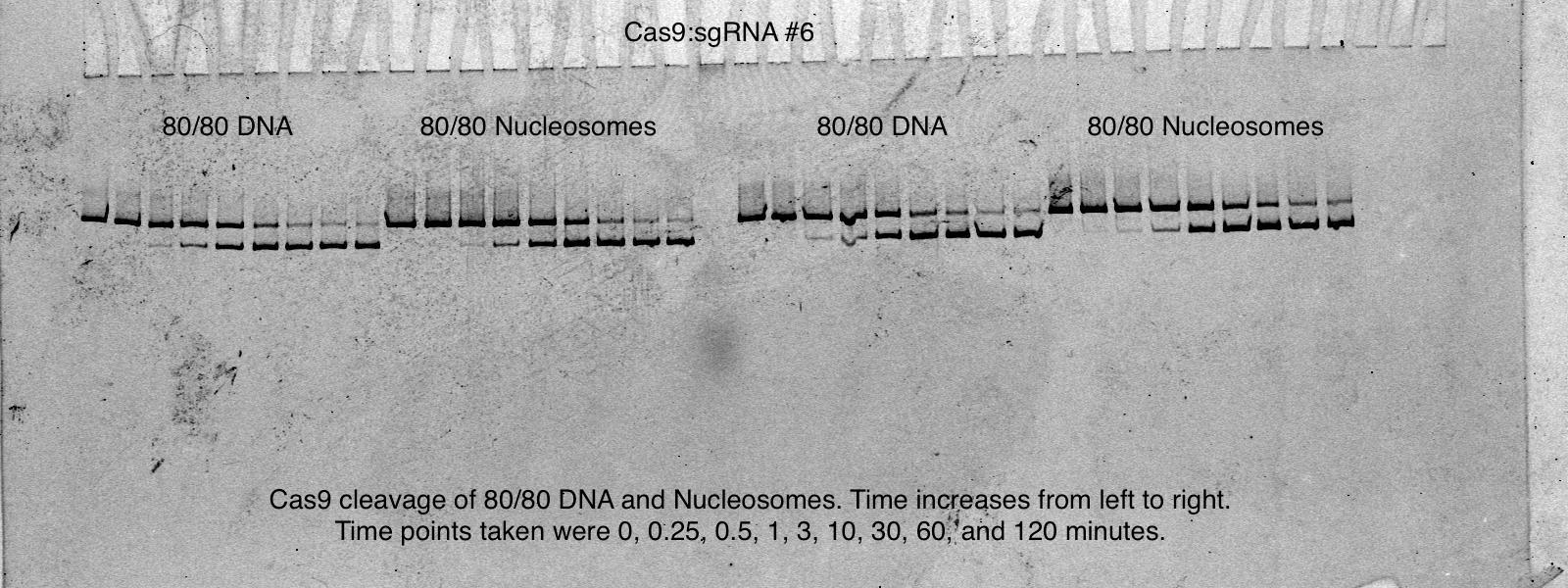

Supplement: Figure 1—source data 3. — DOI: http://dx.doi.org/10.7554/eLife.13450.006 [file elife-13450-fig1-data3.zip › Figure_1Source_Data_3.jpg]

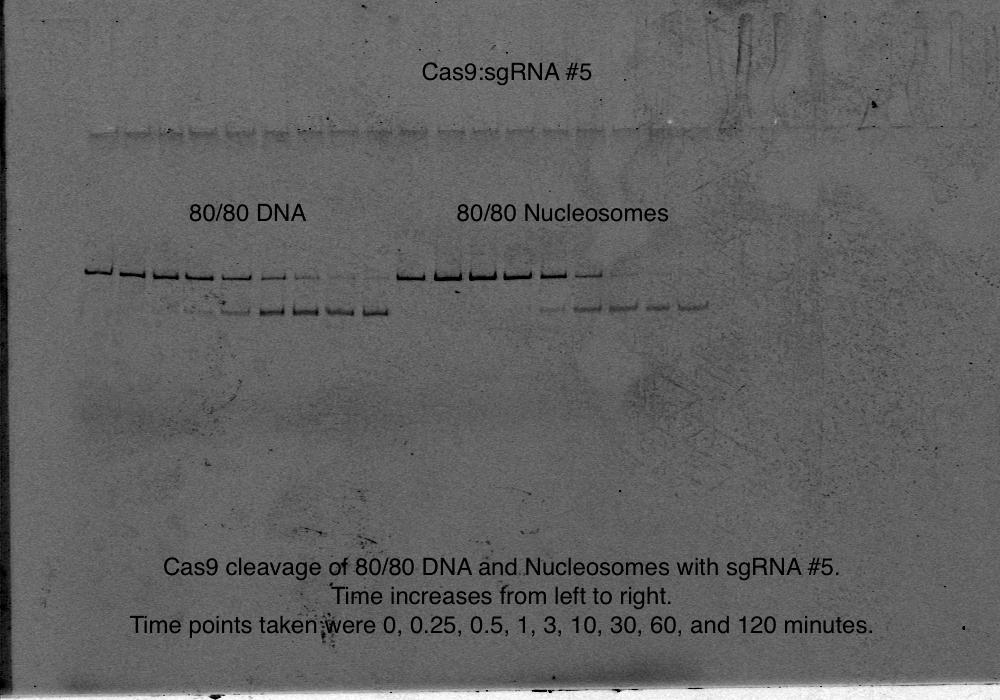

Supplement: Figure 1—source data 4. — DOI: http://dx.doi.org/10.7554/eLife.13450.007 [file elife-13450-fig1-data4.zip › Figure_1Source_Data_4.jpg]

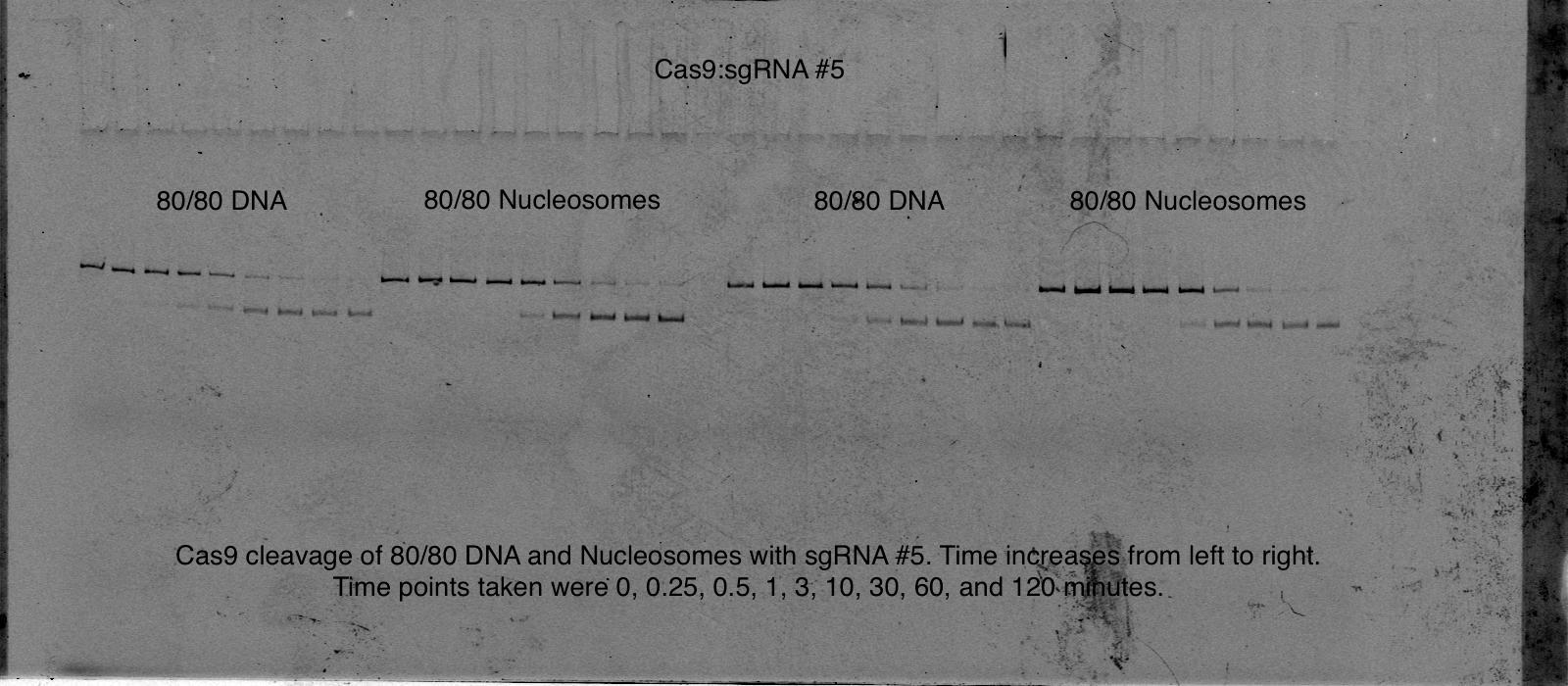

Supplement: Figure 1—source data 5. — DOI: http://dx.doi.org/10.7554/eLife.13450.008 [file elife-13450-fig1-data5.zip › Figure_1Source_Data_5.jpg]

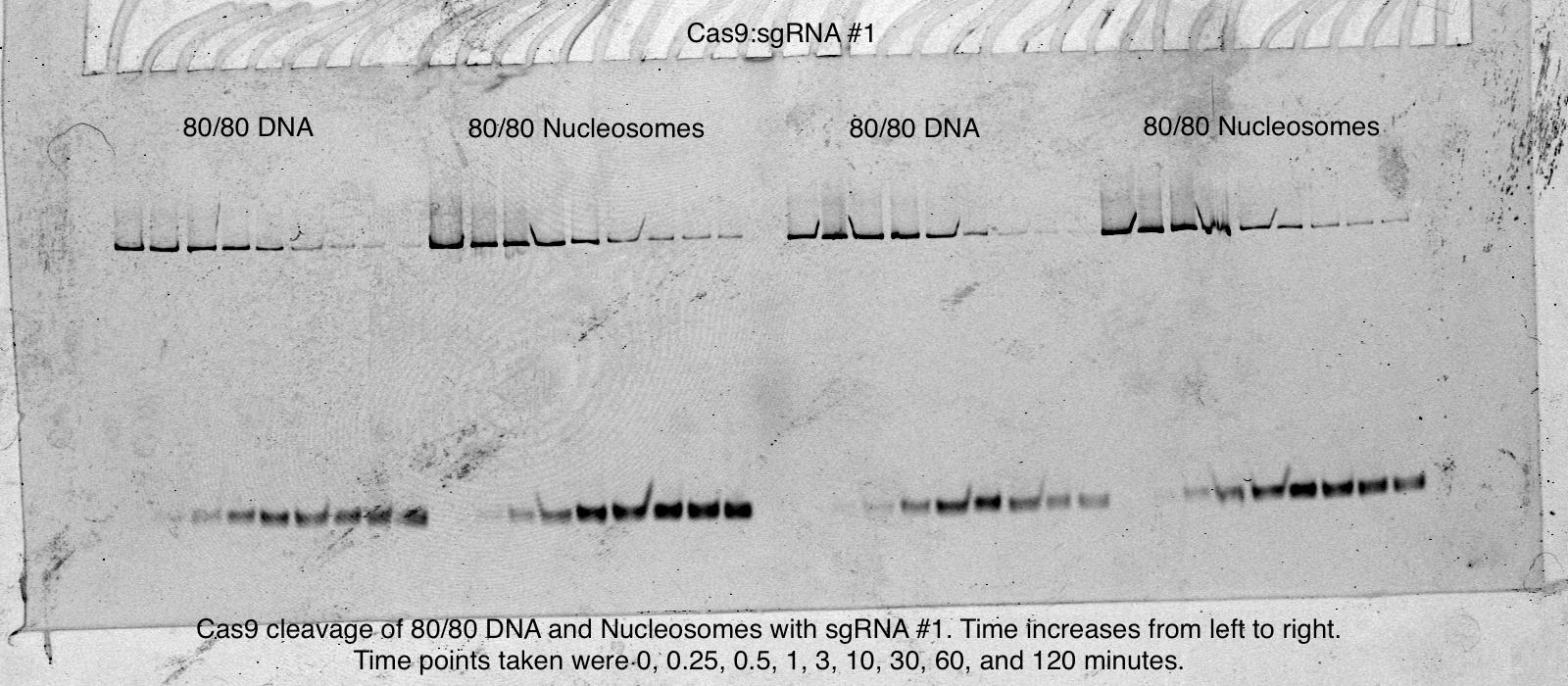

Supplement: Figure 1—source data 6. — DOI: http://dx.doi.org/10.7554/eLife.13450.009 [file elife-13450-fig1-data6.zip › Figure_1Source_Data_6.jpg]

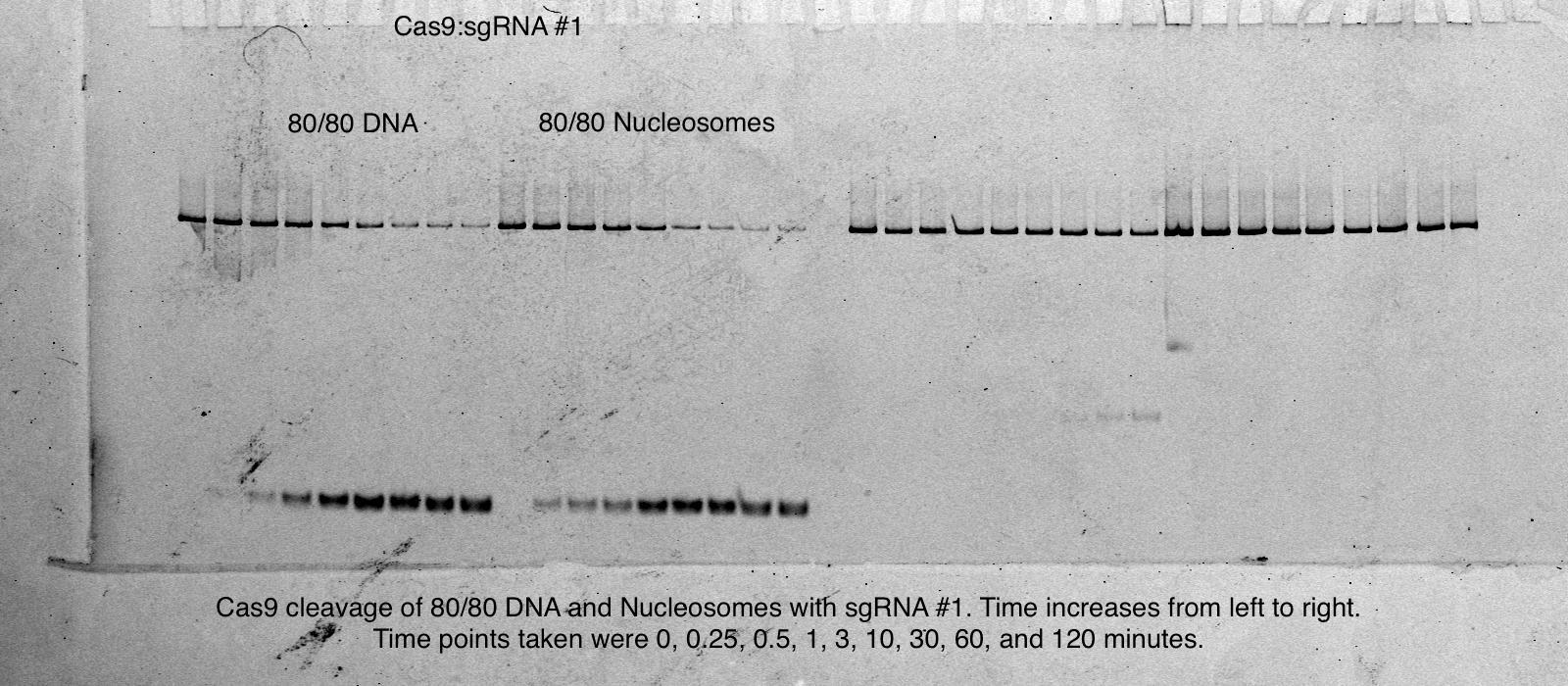

Supplement: Figure 1—source data 7. — DOI: http://dx.doi.org/10.7554/eLife.13450.010 [file elife-13450-fig1-data7.zip › Figure_1Source_Data_7.jpg]

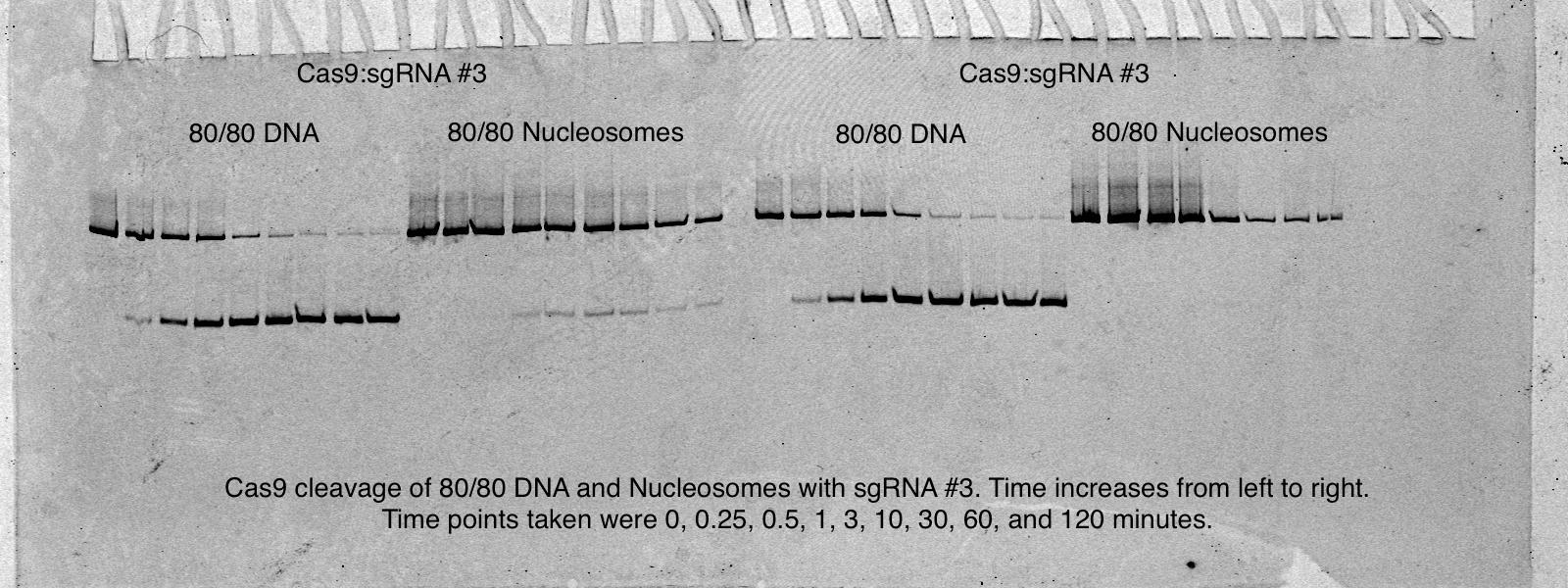

Supplement: Figure 1—source data 8. — DOI: http://dx.doi.org/10.7554/eLife.13450.011 [file elife-13450-fig1-data8.zip › Figure_1Source_Data_8.jpg]

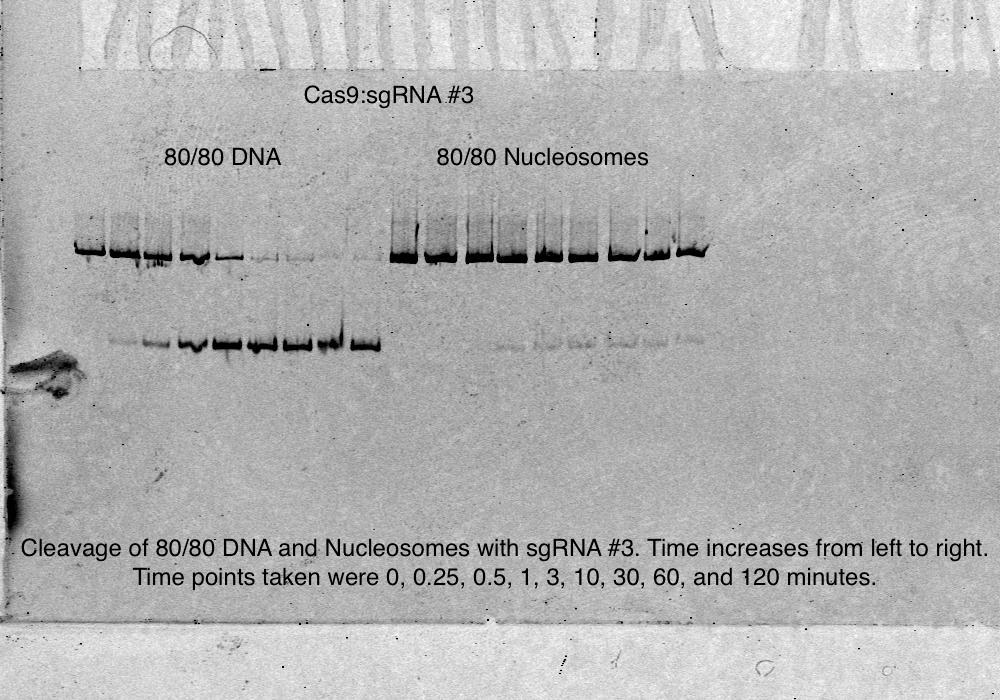

Supplement: Figure 1—source data 9. — DOI: http://dx.doi.org/10.7554/eLife.13450.012 [file elife-13450-fig1-data9.zip › Figure_1Source_Data_9.jpg]

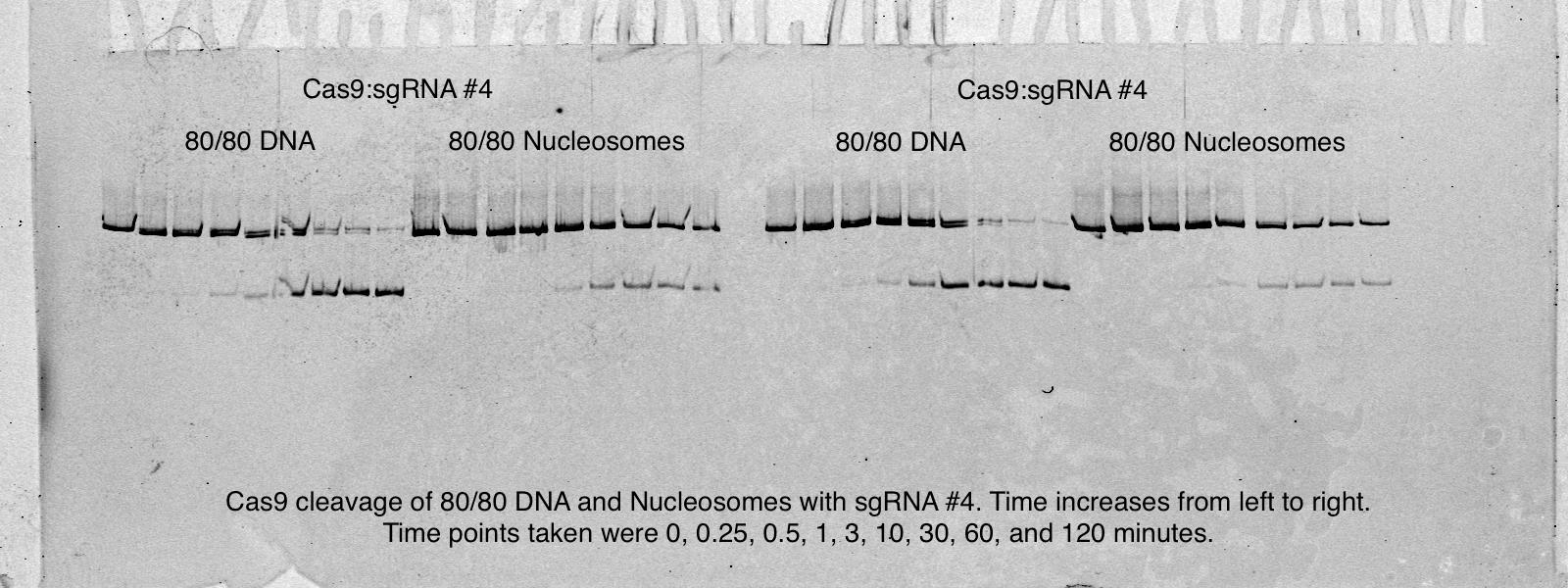

Supplement: Figure 1—source data 10. — DOI: http://dx.doi.org/10.7554/eLife.13450.013 [file elife-13450-fig1-data10.zip › Figure_1Source_Data_10.jpg]

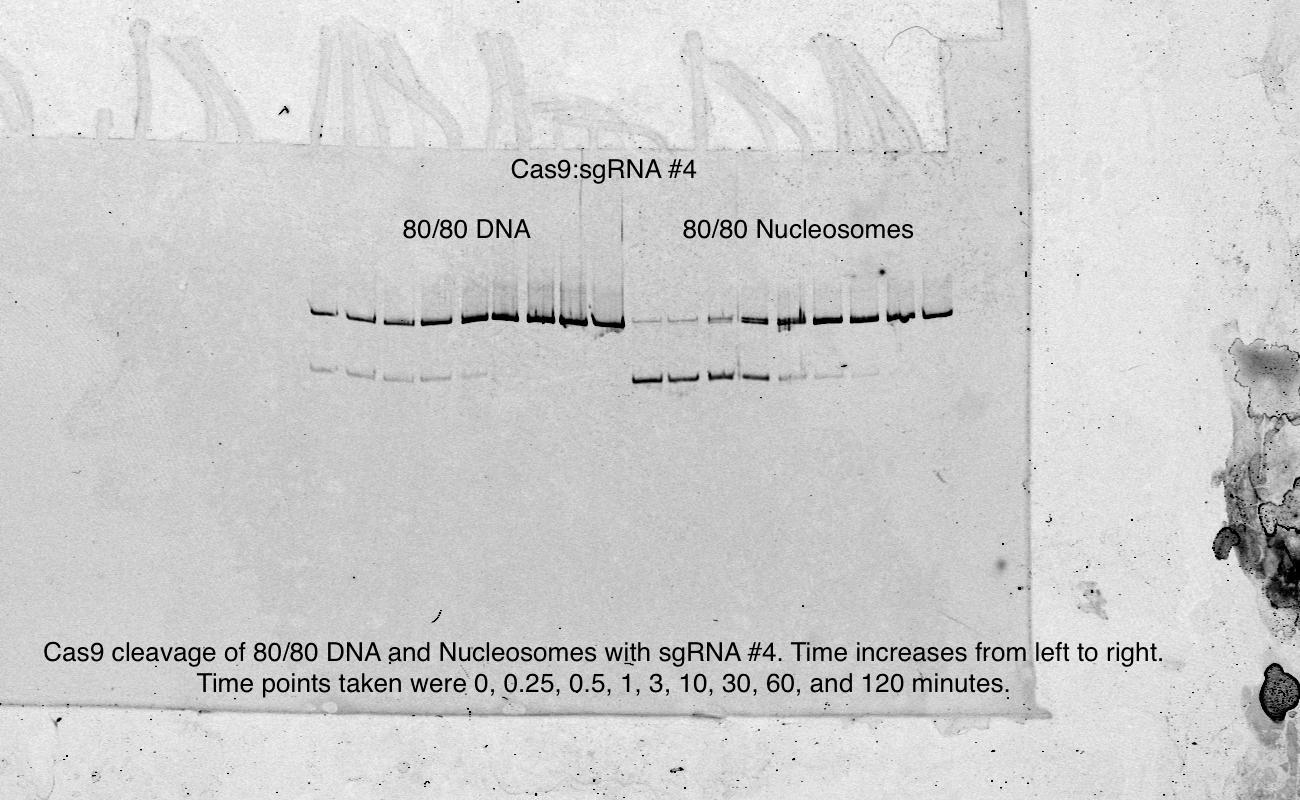

Supplement: Figure 1—source data 11. — DOI: http://dx.doi.org/10.7554/eLife.13450.014 [file elife-13450-fig1-data11.zip › Figure_1Source_Data_11.jpg]

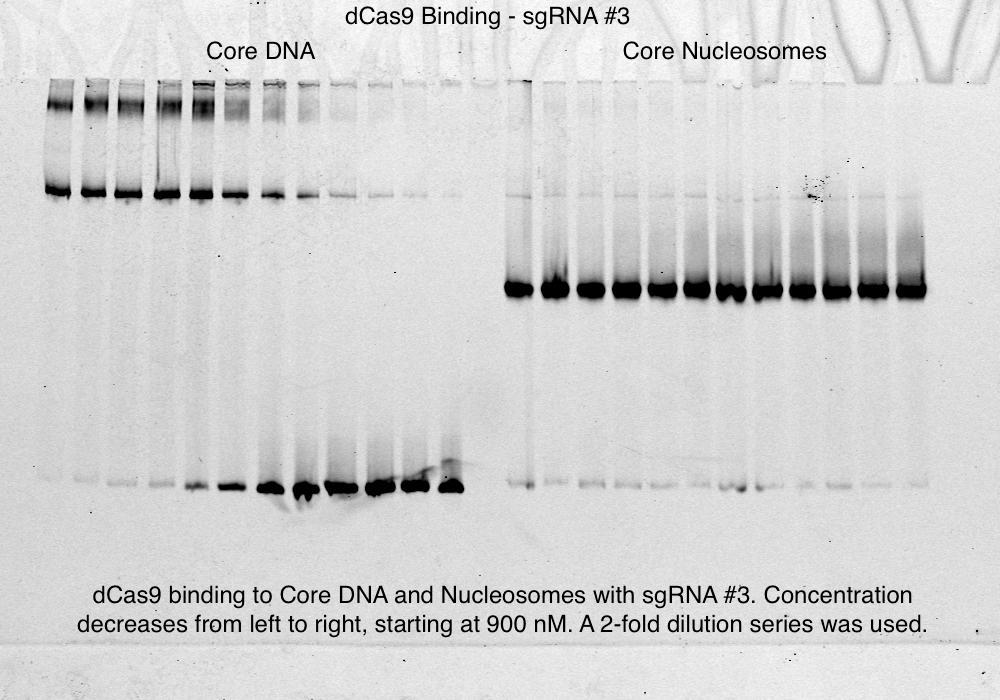

Supplement: Figure 1—figure supplement 1—source data 1. — DOI: http://dx.doi.org/10.7554/eLife.13450.017 [file elife-13450-fig1-figsupp1-data1.zip › Figure_1Supplement_1_Source_Data_1.jpg]

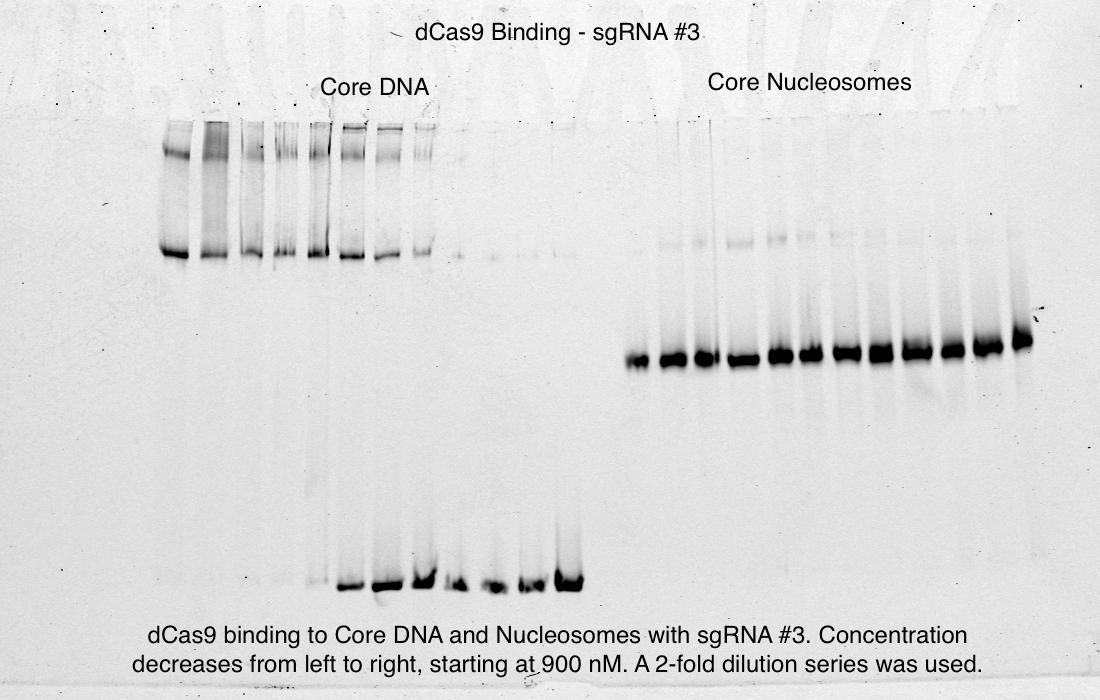

Supplement: ‌‌Figure 1—figure supplement 1—source data 2. — DOI: http://dx.doi.org/10.7554/eLife.13450.018 [file elife-13450.zip › Figure_1Supplement_1_Source_Data_2.jpg]

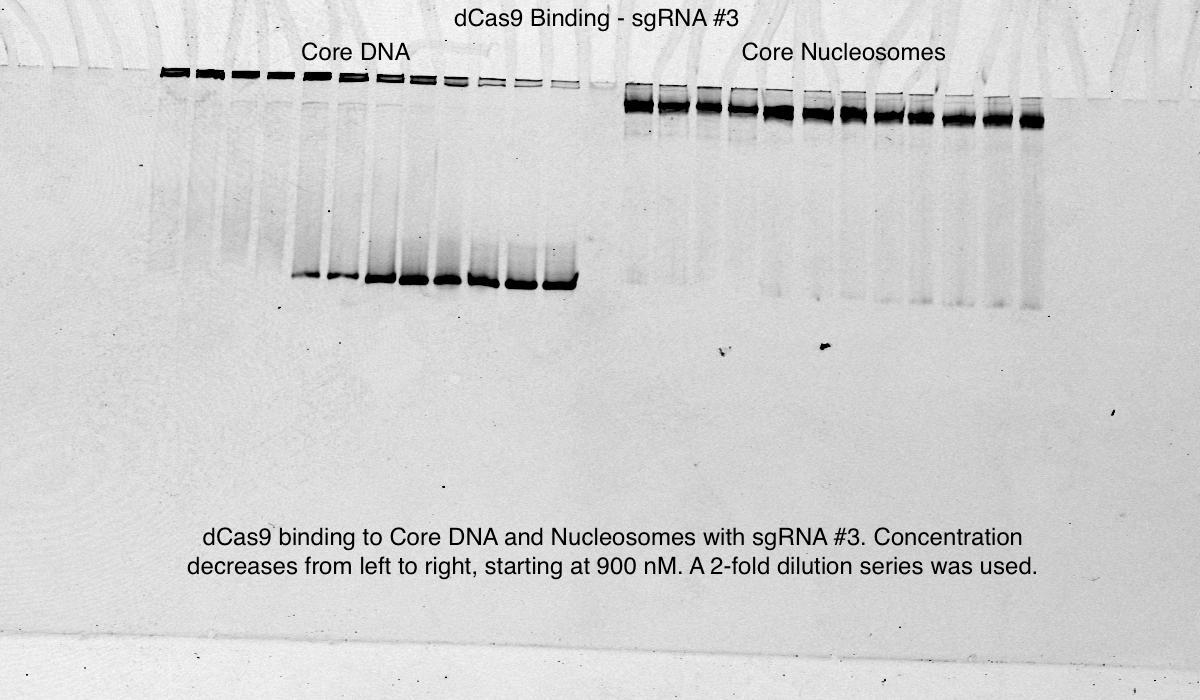

Supplement: Figure 1—figure supplement 1—source data 3. — DOI: http://dx.doi.org/10.7554/eLife.13450.019 [file elife-13450-fig1-figsupp1-data3.zip › Figure_1Supplement_1_Source_Data_3.jpg]

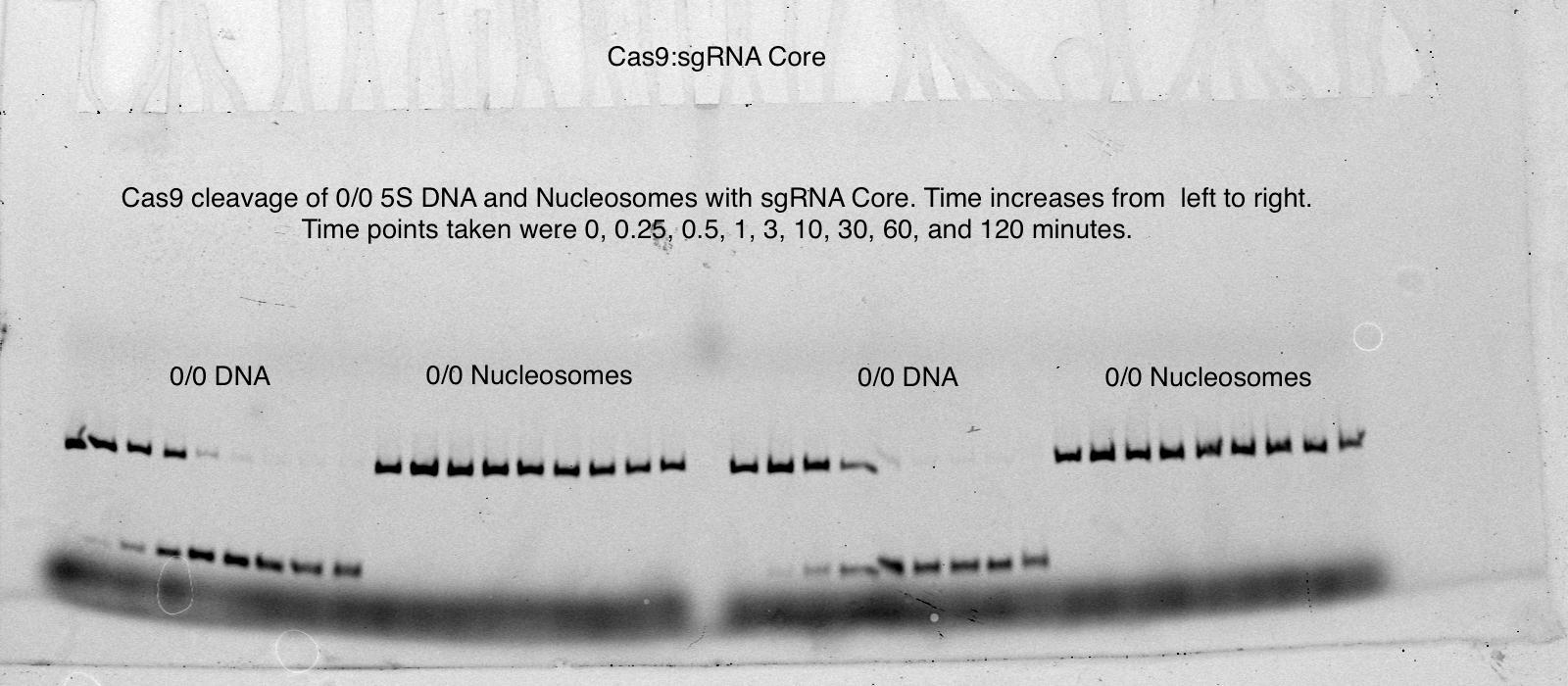

Supplement: Figure 2—source data 1. — DOI: http://dx.doi.org/10.7554/eLife.13450.022 [file elife-13450-fig2-data1.zip › Figure_2Source_Data_1.jpg]

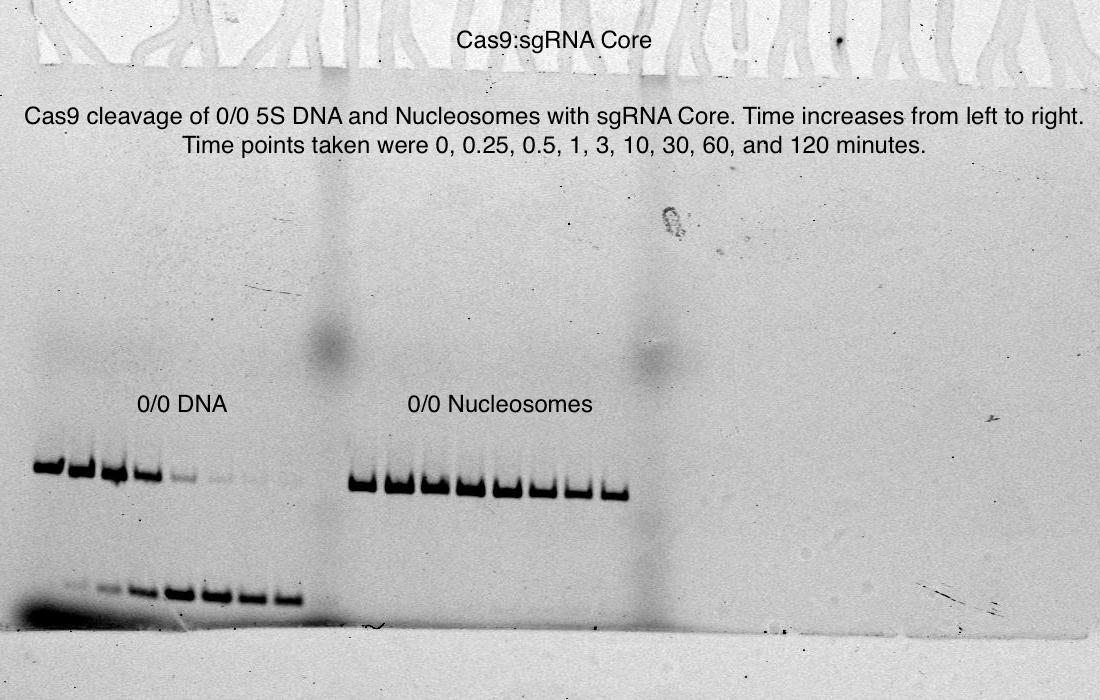

Supplement: Figure 2—source data 2. — DOI: http://dx.doi.org/10.7554/eLife.13450.023 [file elife-13450-fig2-data2.zip › Figure_2Source_Data_2.jpg]

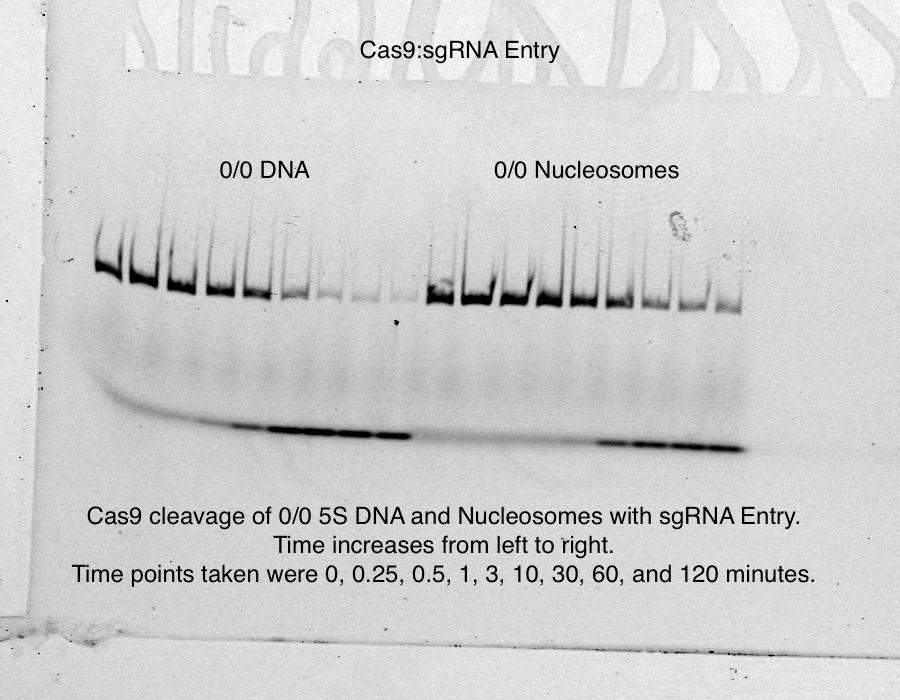

Supplement: Figure 2—source data 3. — DOI: http://dx.doi.org/10.7554/eLife.13450.024 [file elife-13450-fig2-data3.zip › Figure_2Source_Data_3.jpg]

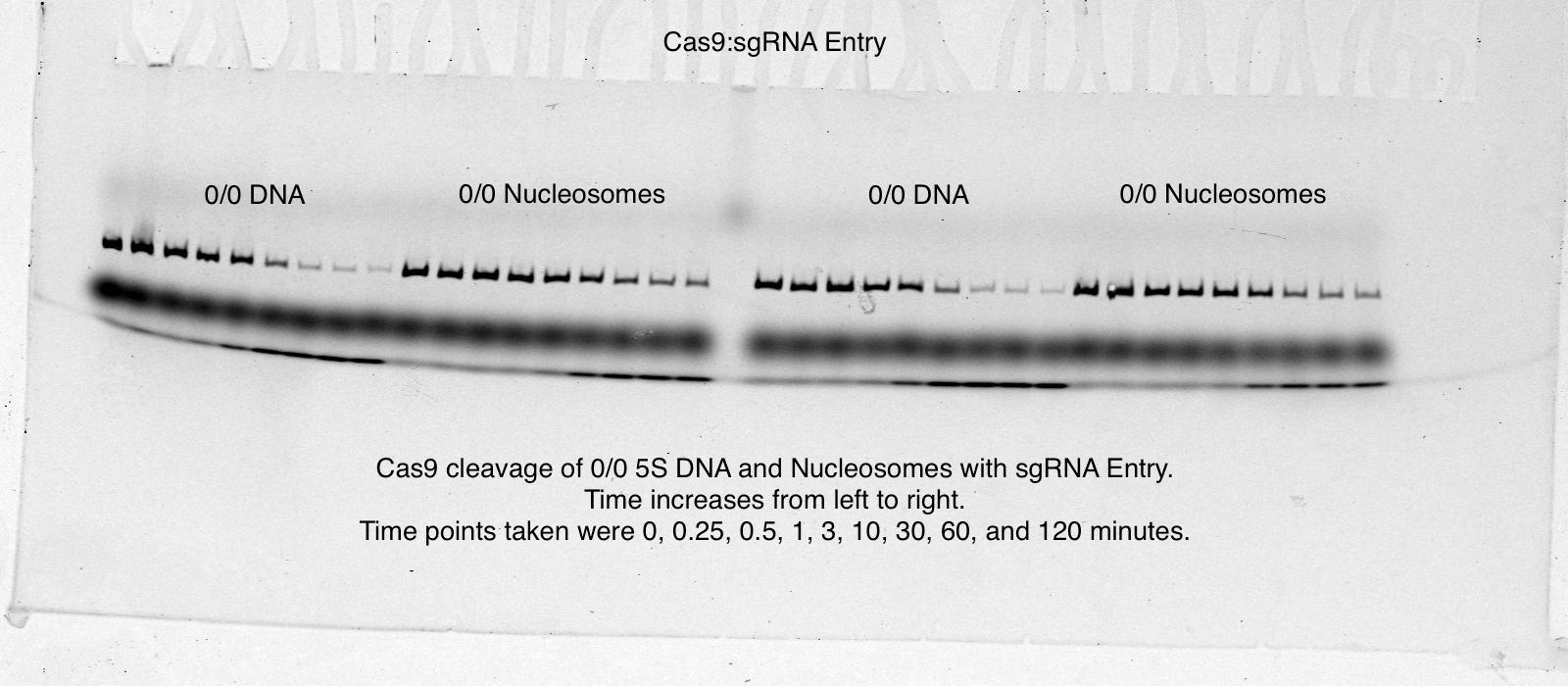

Supplement: Figure 2—source data 4. — DOI: http://dx.doi.org/10.7554/eLife.13450.025 [file elife-13450-fig2-data4.zip › Figure_2Source_Data_4.jpg]

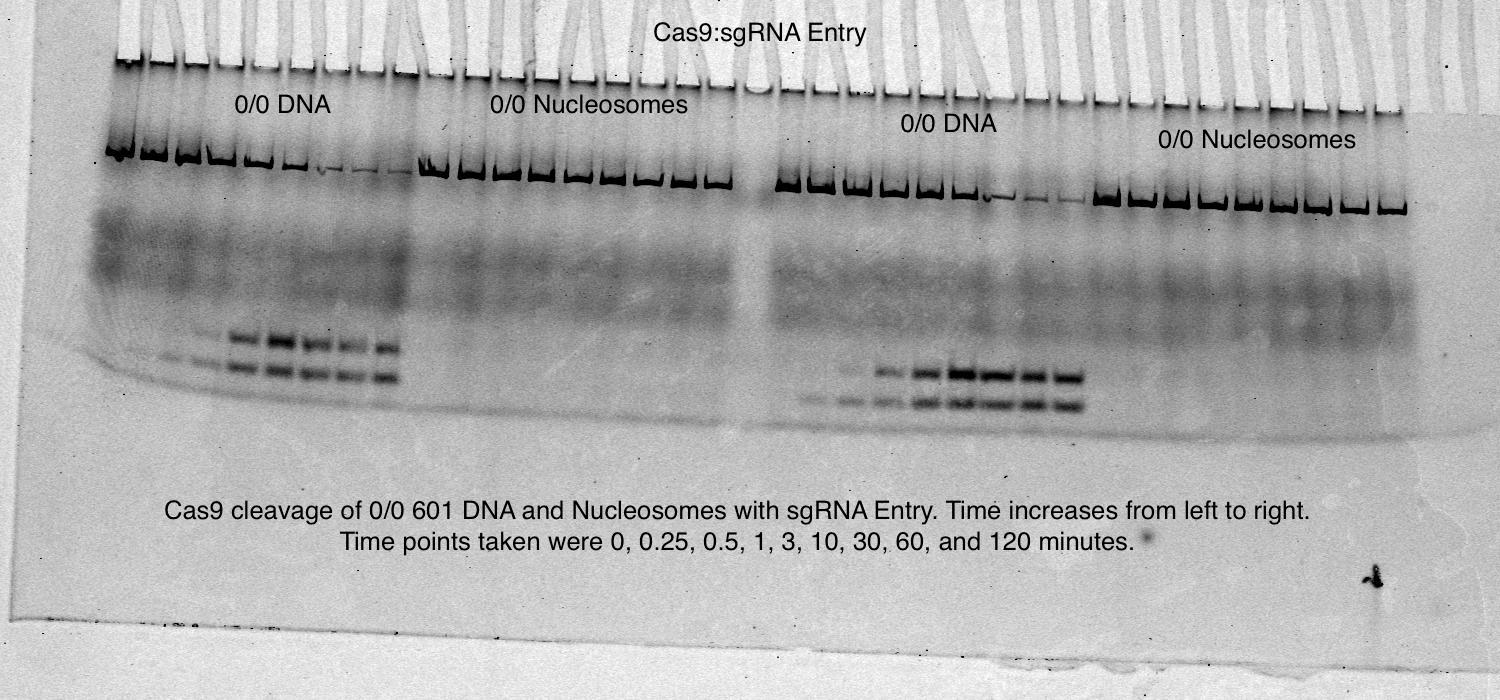

Supplement: Figure 2—source data 5. — DOI: http://dx.doi.org/10.7554/eLife.13450.026 [file elife-13450-fig2-data5.zip › Figure_2Source_Data_5.jpg]

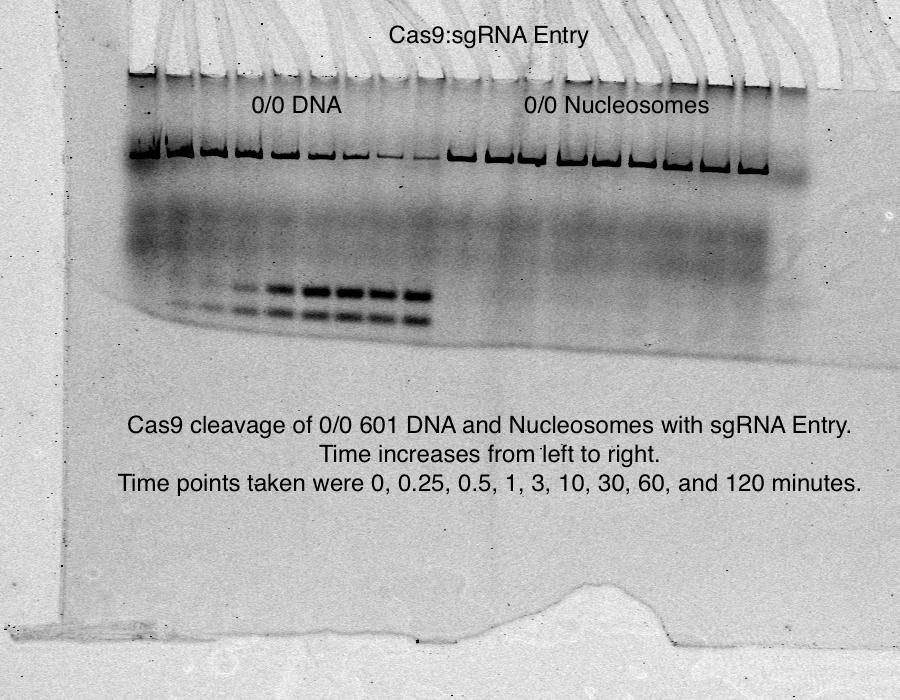

Supplement: Figure 2—source data 6. — DOI: http://dx.doi.org/10.7554/eLife.13450.027 [file elife-13450-fig2-data6.zip › Figure_2Source_Data_6.jpg]

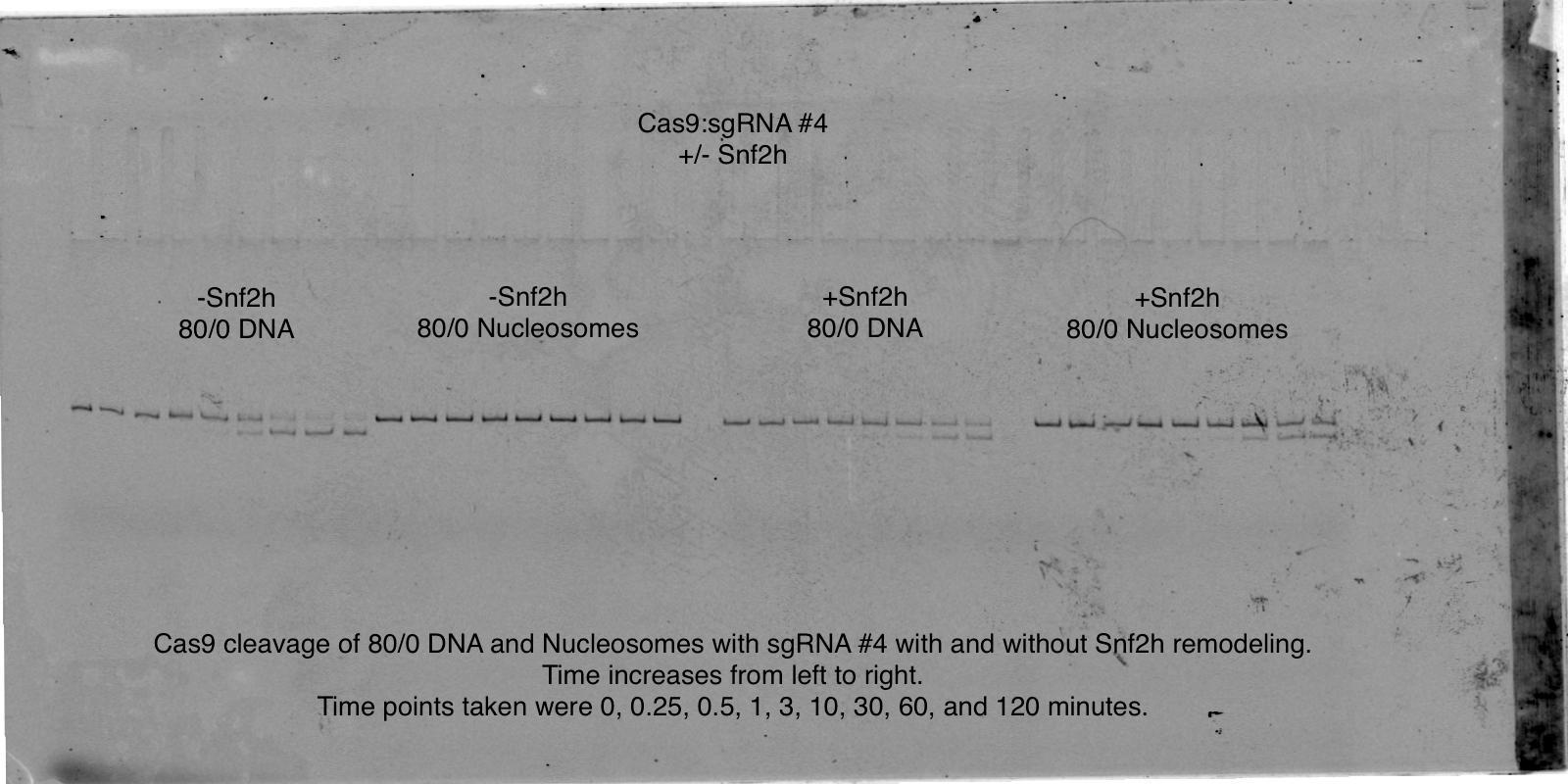

Supplement: Figure 3—source data 1. — DOI: http://dx.doi.org/10.7554/eLife.13450.032 [file elife-13450-fig3-data1.zip › Figure_3Source_Data_1.jpg]

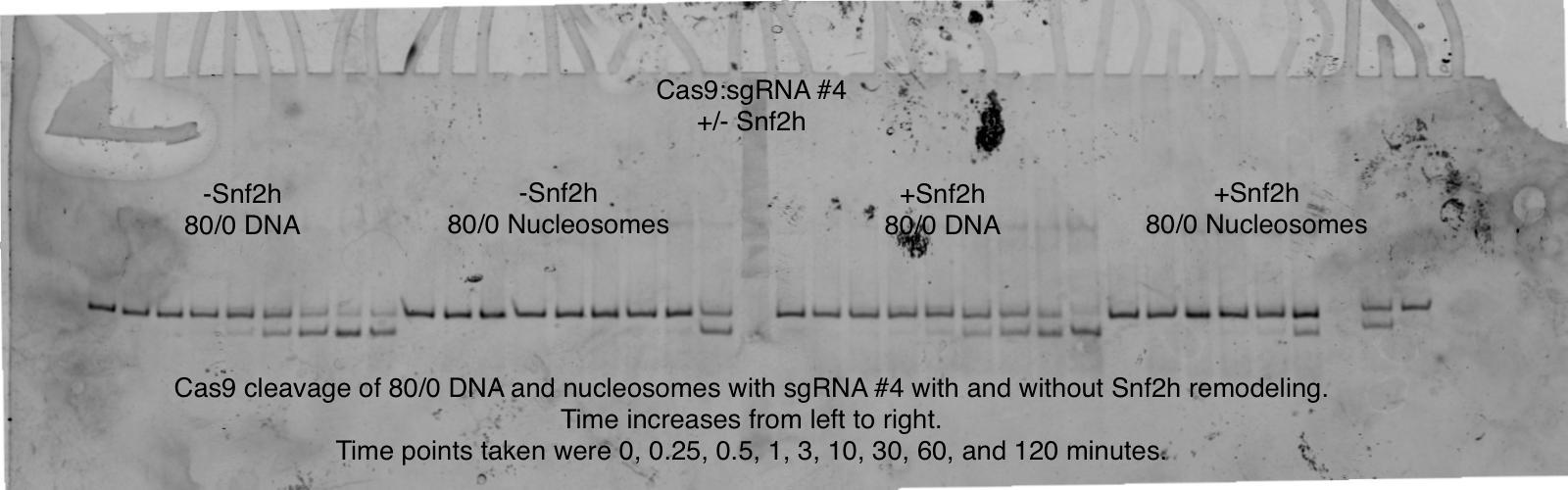

Supplement: Figure 3—source Data 2. — DOI: http://dx.doi.org/10.7554/eLife.13450.033 [file elife-13450-fig3-data2.zip › Figure_3Source_Data_2.jpg]

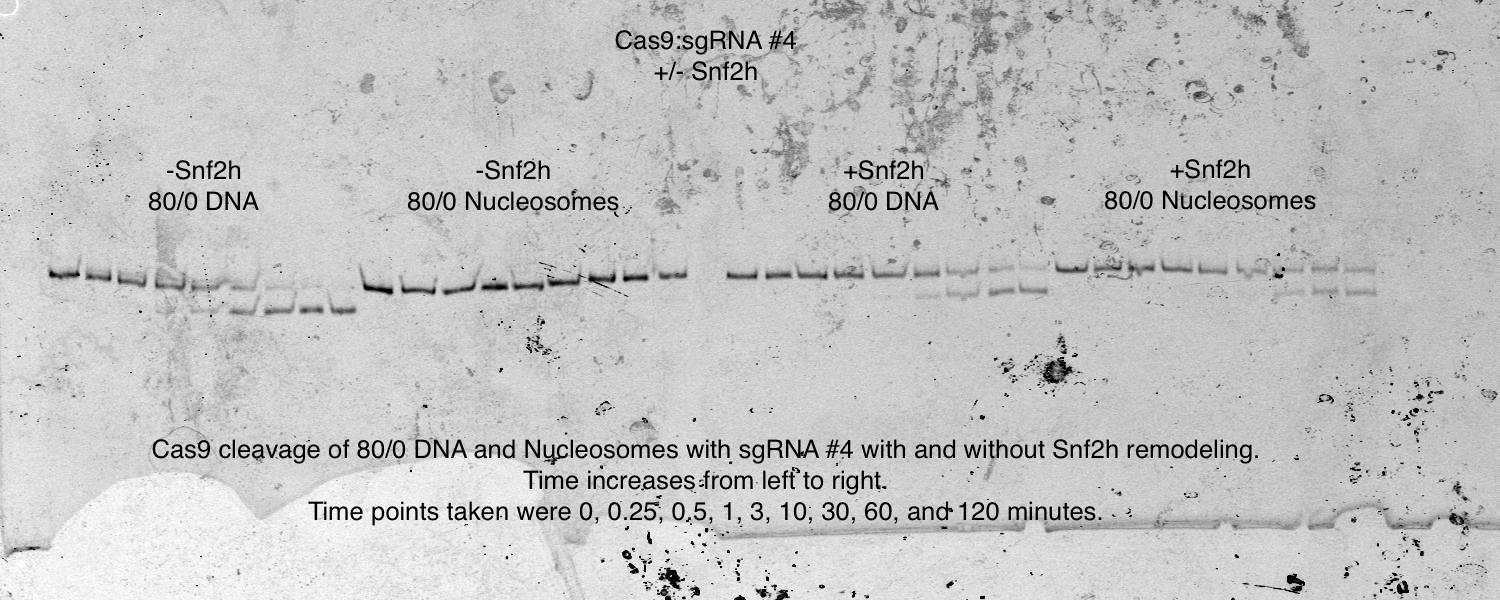

Supplement: Figure 3—source data 3. — DOI: http://dx.doi.org/10.7554/eLife.13450.034 [file elife-13450-fig3-data3.zip › Figure_3Source_Data_3.jpg]

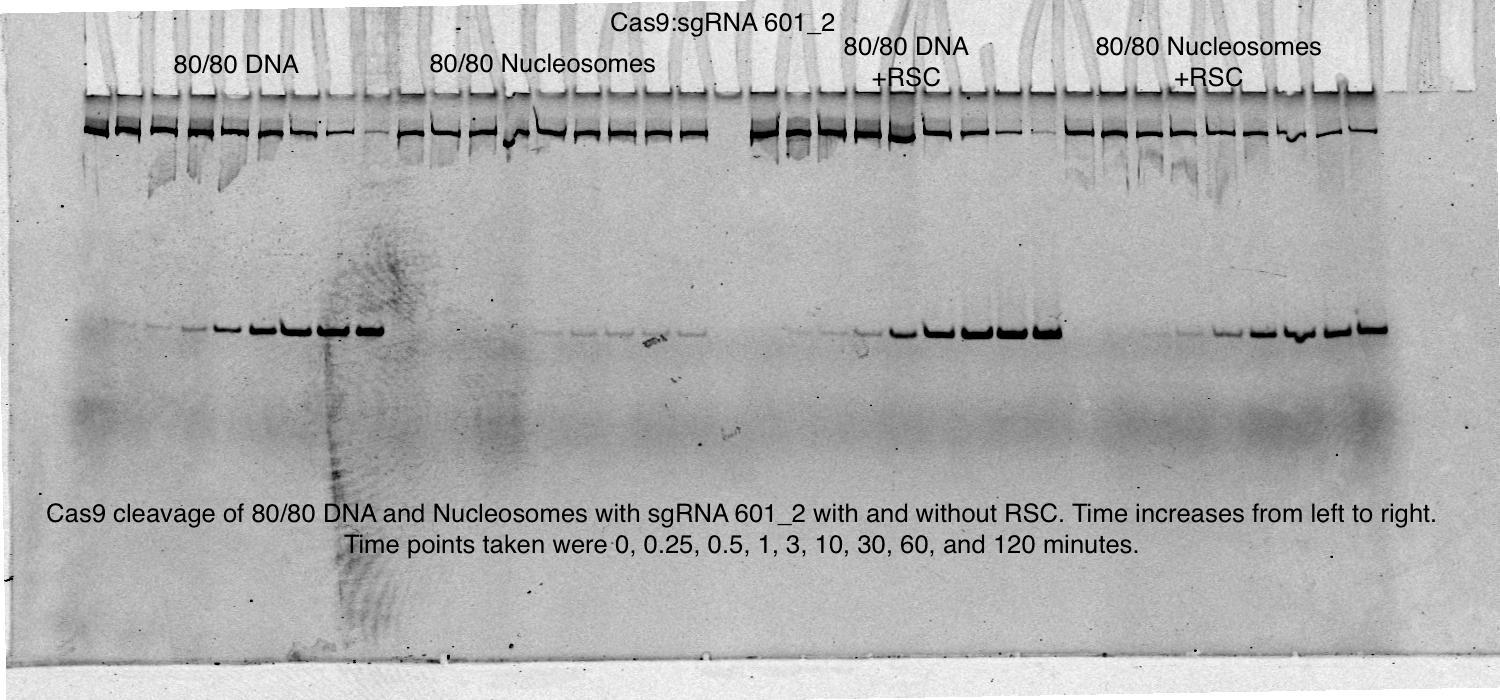

Supplement: Figure 3—source data 5. — DOI: http://dx.doi.org/10.7554/eLife.13450.036 [file elife-13450-fig3-data5.zip › Figure_3Source_Data_5.jpg]

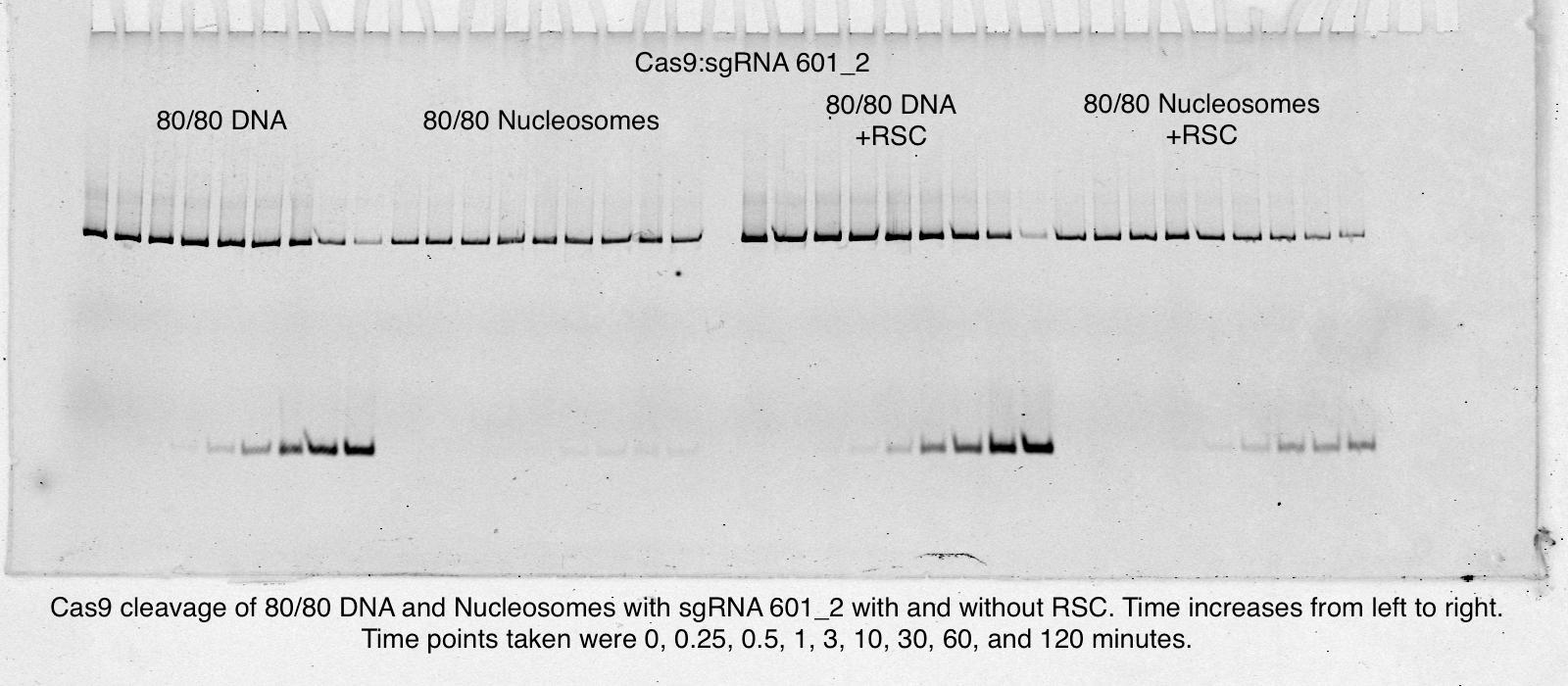

Supplement: Figure 3—source data 6. — DOI: http://dx.doi.org/10.7554/eLife.13450.037 [file elife-13450-fig3-data6.zip › Figure_3Source_Data_6.jpg]

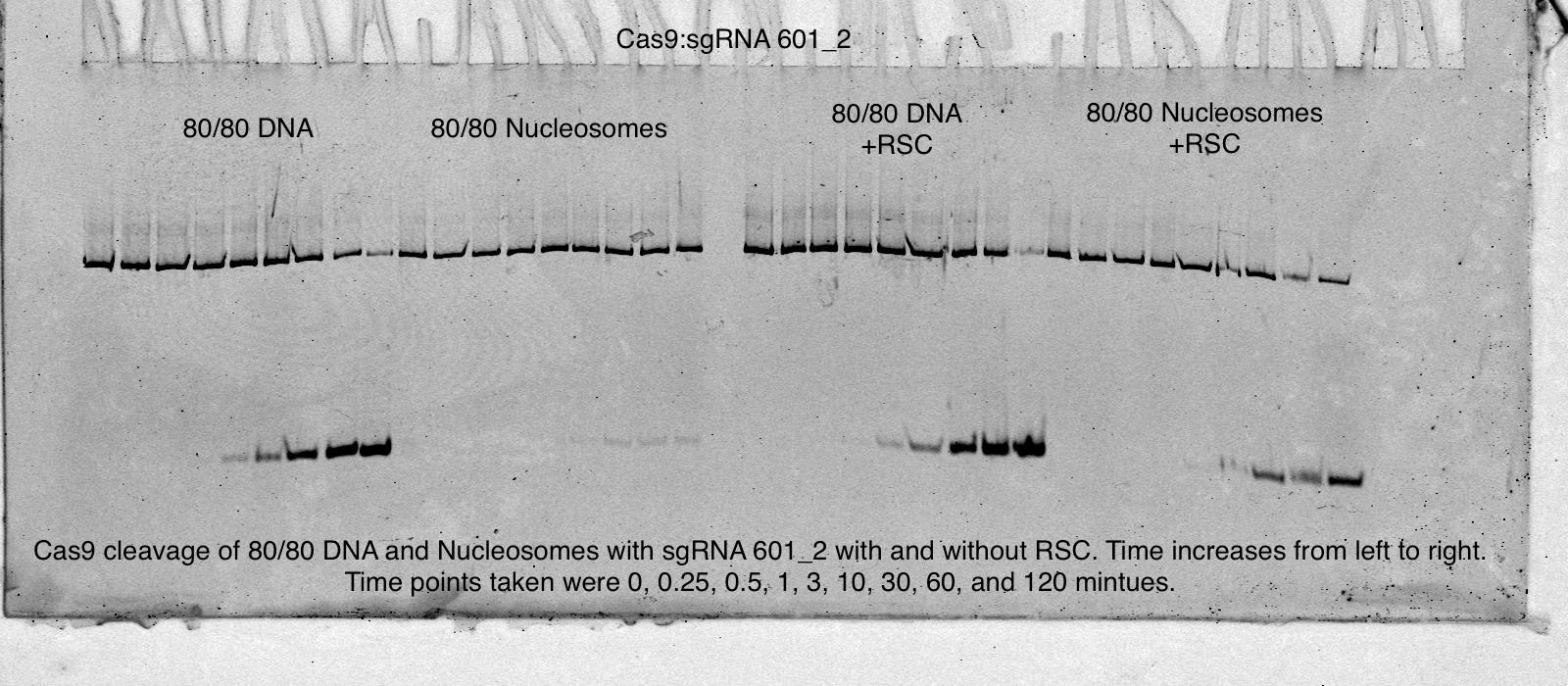

Supplement: Figure 3—source data 7. — DOI: http://dx.doi.org/10.7554/eLife.13450.038 [file elife-13450-fig3-data7.zip › Figure_3Source_Data_7.jpg]

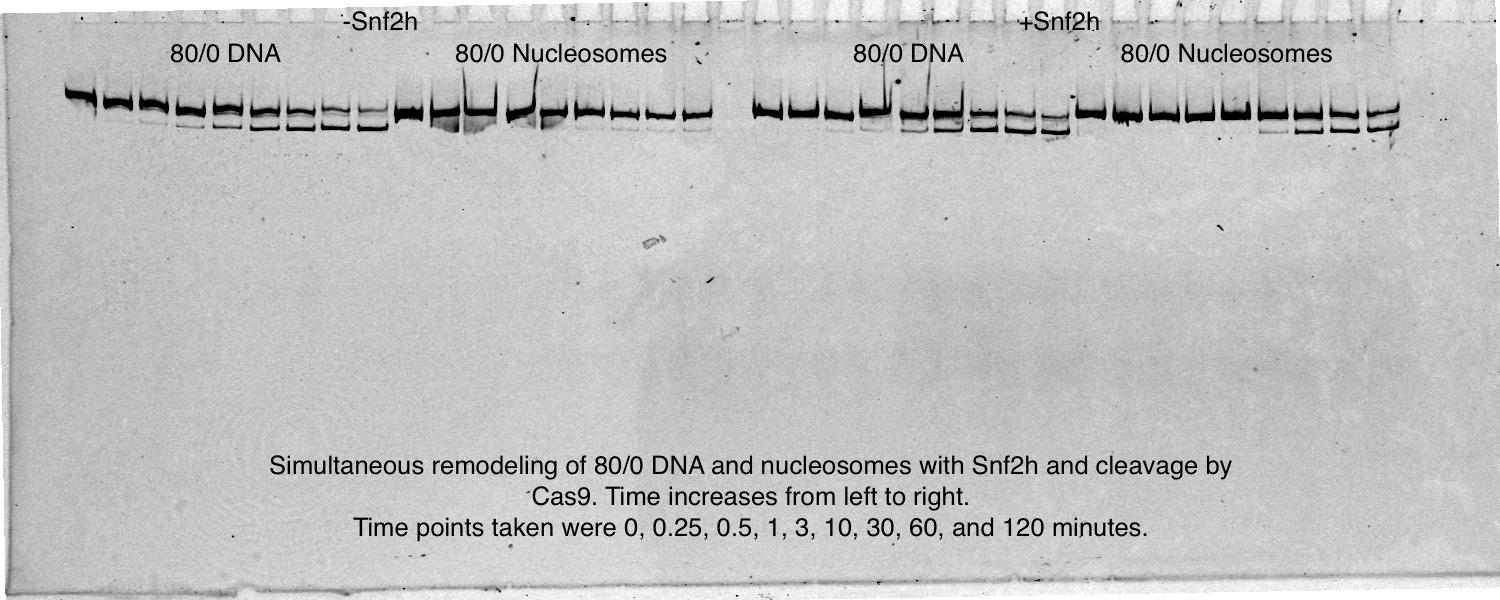

Supplement: Figure 3—figure supplement 2—source data 1. — DOI: http://dx.doi.org/10.7554/eLife.13450.042 [file elife-13450-fig3-figsupp2-data1.zip › Figure_3Supplement_2_Source_Data_1.jpg]

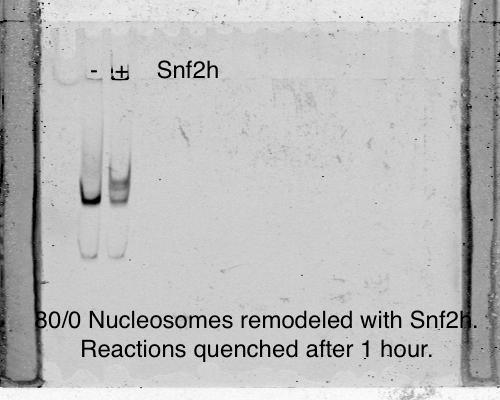

Supplement: Figure 3—figure supplement 3—source data 1. — DOI: http://dx.doi.org/10.7554/eLife.13450.044 [file elife-13450-fig3-figsupp3-data1.zip › Figure_3Supplement_3_Source_Data_1.jpg]

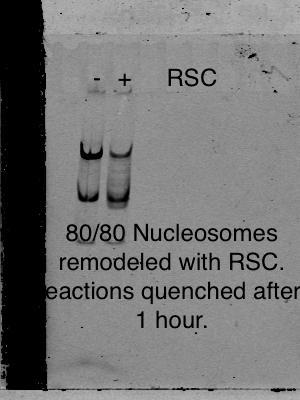

Supplement: Figure 3—figure supplement 3—source data 2. — DOI: http://dx.doi.org/10.7554/eLife.13450.045 [file elife-13450-fig3-figsupp3-data2.zip › Figure_3Supplement_3_Source_Data_2.jpg]
